# Effectiveness of resistance training in combination with botulinum toxin-A on hand and arm use in children with cerebral palsy: a pre-post intervention study

**DOI:** 10.1186/1471-2431-12-91

**Published:** 2012-07-02

**Authors:** Ann-Kristin G Elvrum, Siri M Brændvik, Rannei Sæther, Torarin Lamvik, Beatrix Vereijken, Karin Roeleveld

**Affiliations:** 1 The Clinical Services, St. Olavs University Hospital, Olav Kyrresgt. 17, N-7006, Trondheim, Norway; 2 Department of Human Movement Science, NTNU, Trondheim, Norway; 3 Orthopaedic Department, St. Olavs University Hospital, Trondheim, Norway

**Keywords:** Cerebral palsy, Hand and arm use, Botulinum Toxin-A, Strength training

## Abstract

**Background:**

The aim of this pilot study was to examine the effects of additional resistance training after use of Botulinum Toxin-A (BoNT-A) on the upper limbs in children with cerebral palsy (CP).

**Methods:**

Ten children with CP (9–17 years) with unilaterally affected upper limbs according to Manual Ability Classification System II were assigned to two intervention groups. One group received BoNT-A treatment (group B), the other BoNT-A plus eight weeks resistance training (group BT). Hand and arm use were evaluated by means of the Melbourne assessment of unilateral upper limb function (Melbourne) and Assisting Hand Assessment (AHA). Measures of muscle strength, muscle tone, and active range of motion were used to assess neuromuscular body function. Measurements were performed before and two and five months after intervention start. Change scores and differences between the groups in such scores were subjected to Mann–Whitney U and Wilcoxon Signed Rank tests, respectively.

**Results:**

Both groups had very small improvements in AHA and Melbourne two months after BoNT-A injections, without differences between groups. There were significant, or close to significant, short-term treatment effects in favour of group BT for muscle strength in injected muscles (elbow flexion strength, *p* = .08) and non-injected muscles (elbow extension and supination strength, both *p* = .05), without concomitant increases in muscle tone. Active supination range improved in both groups, but more so in group BT (*p* = .09). There were no differences between the groups five months after intervention start.

**Conclusions:**

Resistance training strengthens non-injected muscles temporarily and may reduce short-term strength loss that results from BoNT-A injections without increasing muscle tone. Moreover, additional resistance training may increase active range of motion to a greater extent than BoNT-A alone. None of the improvements in neuromuscular impairments further augmented use of the hand and arm. Larger clinical trials are needed to establish whether resistance training can counteract strength loss caused by BoNT-A, whether the combination of BoNT-A and resistance training is superior to BoNT-A or resistance training alone in improving active range of motion, and whether increased task-related training is a more effective approach to improve hand and arm use in children with CP.

## Background

Cerebral palsy (CP) is the most common cause of physical disability in childhood, with a prevalence of about 2.1 per 1000 live births [1]. Among these children, between 50–70% have impaired upper limb function [2-4]. According to the International Classification of Functioning, Disability and Health (ICF) framework [5], the causes and consequences of impaired upper limb function can be assessed at the levels of body function, activity, and participation. Spasticity, muscle weakness and impaired motor control are primary impairments at the level of body function for children with hemiplegic or diplegic CP. The primary impairments may give rise to secondary musculoskeletal complications such as contractures and deformities, again resulting in limited range of motion [6]. Activity limitations are presumed to result from these motor impairments and also from additional disturbances of sensation, perception, cognition, communication, and behaviour [7]. Spasticity, reduced muscle strength, and limited active supination range have been found to be related to hand and arm use [8], which may lead to difficulties performing several activities of daily living involving reaching, grasping, and manipulating objects. Consequently, much of the treatment offered is targeted towards treating neuromuscular impairments at the level of body functions, while intending to improve performance at the level of activity [9]. However, there is a lack of evidence as to what extent these treatments alter the motor prognosis or make a clinically significant change in hand and arm use at the level of activity in ICF [10,11].

Injections of Botulinum Toxin-A (BoNT-A) are often applied to improve impairments at the level of body functions, such as reducing spasticity, facilitating movement, and preventing secondary contractures, and ultimately improve hand and arm use in children with spastic CP [9]. The beneficial effect of BoNT-A injections in spastic lower limbs on gait has been well documented [12], but there is insufficient evidence with respect to improvements in hand and arm use [12,13]. A recent systematic review concluded that there is moderate evidence that BoNT-A injections alone are not effective in this respect and need to be combined with therapy to obtain functional gains [14]. However, the most effective treatment combinations remain unclear. BoNT-A in combination with resistance training is one treatment combination that warrants further investigation [14]. A few studies have investigated the combined effect of BoNT-A and intensive therapy with strength training as part of the intensive therapy [e.g., [15-17]]. However, these did not describe the procedures or measure the effect of strength training explicitly, making it difficult to evaluate whether such training has the potential to enhance the outcomes of BoNT-A injections. Previously it has been argued that only antagonists to spastic muscles should be strengthened in the BoNT-A effect period, during which spastic agonists have reduced muscle tone, in order to improve muscle balance across joints [9,18]. However, spastic muscles in the upper limbs have been found to show reduced strength during voluntary activation as well [19,20]. In addition, excessive weakness in injected spastic muscles is the most common adverse effect following BoNT-A treatment in the upper limbs [14]. Consequently, both the spastic muscles and their antagonists should be strengthened.

The objective of this pilot study was to explore the effects of additional resistance training of spastic muscles and their antagonists on hand and arm use and neuromuscular body functions in children with CP after the use of BoNT-A. Hand and arm use were evaluated both according to capacity (what the child can do) with Melbourne assessment of unilateral upper limb function (Melbourne) [21,22], and according to performance (what the child spontaneously does) with Assisting Hand Assessment (AHA) [22,23]. Neuromuscular body functions were evaluated by testing active range of motion, muscle strength and muscle tone in spastic muscles and their antagonists in children with CP.

## Methods

### Participants

Participants were recruited from the outpatient records of the neuro-orthopaedic team at St. Olav’s Hospital (Trondheim, Norway). Children with CP were eligible to participate if they had: a) larger deficits in movement of one upper extremity in comparison to the other, b) functional use of the hands corresponding to Manual Ability Classification System (MACS) levels I or II [24], and an active grasp function in the involved extremity corresponding to category 5 (fair active assist) on the Modified House Functional Classification System [25], c) functional limitations when using the involved arm because of increased muscle tone in the forearm and elbow flexor muscles and a difference in active and passive range of motion, and d) were able to follow instructions and motivated to complete an eight week intensive resistance training program. Exclusion criteria were: a) treatment with BoNT-A in the upper extremities during the last six months, b) surgery on the upper extremity in the last two years prior to participation, and c) on-going intensive training.

Thirty-one children were assessed and eighteen were found to be eligible to participate in the study. Eight of the eighteen refused to participate because they did not want BoNT-A injections. The remaining ten participants comprised nine children with hemiplegic and one with diplegic CP, all with MACS level II. These were matched in pairs based on age and assigned through tossing a coin to either a group receiving localized BoNT-A injections (group B, n = 5) or to a group receiving localized BoNT-A injections and resistance training (group BT, n = 5). Table 1 shows the demographic data for each of the groups.

**Table 1:** Demographic data for the groups

	**Group B (n = 5)**	**Group BT (n = 5)**
Mean age (SD)	12 years (3.3 years)	14.8 years (3.0 years)
Male/female (n)	4/1	1/4
CP type, hemiplegic/diplegic(n)	4/1	5/0

Group B = Botulinum Toxin-A injections.

Group BT = Botulinum Toxin-A injections + eight weeks of resistance training.

n = number of participants.

SD = Standard deviation.

The study was approved by the Regional Committee for Medical and Health Research Ethics. Written informed consent was obtained from the participants and parents before participation.

Data were collected from September 2006 until March 2008. Participants were assessed on three occasions: before intervention (Baseline) and at two months (Post 2 months) and five months (Post 5 months) after the BoNT-A treatment, whether or not participating in resistance training. Baseline data from the study have been reported in a previous publication [8].

### Interventions

All the participants were instructed to continue their usual daily activities during the study period. None of the participants in group B performed specific training related to hand function, whereas those in group BT performed single-joint resistance training without additional functional training.

All participants in groups B and BT received BoNT-A injections in m. pronator teres. In addition, three participants in group B and two in group BT received BoNT-A injections in m. biceps brachii and m. brachialis. Details of BoNT-A treatment are listed in Table 2. No participants received injections in spastic muscles in the hand or fingers because we wanted to avoid potential loss of grip strength [26] that might influence the possibility to perform the resistance training. BOTOX® from Allergan (Irvine, CA) was used (dilution 100 U/1 mL) and dosage was 25 or 50 U per injection sites, one site in pronator teres and brachialis, and two sites in biceps brachii. An experienced physician who was blinded for group assignment decided the dosage and performed the injections under general anaesthesia. A muscle stimulator was used in order to optimize accuracy of the injections of pronator teres [27]. 

**Table 2:** Details of the Botulinum Toxin-A (BoNT-A) treatment for the participants in groups B and BT

**Participant**	**Group B**	**Group BT**
	**Muscle groups injected:**	**Muscle groups injected:**
1	Pronator teres	Pronator teres
2	Pronator teres, biceps brachii, brachialis	Pronator teres
3	Pronator teres, biceps brachii, brachialis	Pronator teres, biceps brachii, brachialis
4	Pronator teres, biceps brachii, brachialis	Pronator teres, biceps brachii, brachialis
5	Pronator teres	Pronator teres

Pronator teres 25–50 units BOTOX® (Allergan, Inc.).

Biceps brachii 50–75 units BOTOX® (Allergan, Inc.).

Brachialis 25–50 units BOTOX® (Allergan, Inc.).

The participants in group BT trained three times a week for eight weeks under supervision of a trained physiotherapist not involved in the assessments. Participants exercised alone or in pairs. The resistance training program followed guidelines from the National Strength and Conditioning Association [28] and consisted of ten minutes warm up and 30–40 minutes core strengthening and single-joint resistance training (using hand-held, free weights) for strengthening of elbow flexors and extensors, forearm pronators and supinators, and wrist flexors and extensors. Grip force was trained as well using exercise balls with increasing resistance. The intensity was set on the basis of 10 repetition maximum strength tested at baseline [29]. Once the participants were able to perform three sets of ten repetitions in an exercise, intensity was built up progressively on an individualized basis by increasing the weights by 0.25 – 0.5 kg. There was a one-minute recovery period between all sets and a two-minute recovery period between different exercises.

### Outcome measures

Hand and arm use were evaluated with Melbourne [21] and with AHA [23]. Melbourne measures unimanual capacity in tasks that simulate everyday activities. The children are asked to perform their best in 16 tasks that are scored according to 37 sub-scores. Percentage scores (0–100) were calculated and used for analyses [21]. Melbourne has been found to be a reliable and valid instrument for measuring quality of upper limb function, and the smallest detectable difference to measure real change needs to be at least 12% [30]. A randomized selection of 3–15 items from seven different subjects was scored by a second therapist. The intra-class correlation coefficient for the consistency of scorings between therapists was high (ICC = 0.96). AHA is a Rasch-built instrument that measures how children spontaneously use their involved upper limb in bimanual tasks (i.e. bimanual performance) without instructing the child to perform at their best [23]. The AHA was scored on 22 items with a four-point rating scale (22–88 raw sore). Scores on AHA were converted into equal interval logits (log odds probability units), which were converted to a 0–100 AHA-unit scale [31]. The smallest detectable difference for AHA is 5 AHA-units [32]. AHA has been found to be valid and reliable [33] and has been validated for children up to 12 years. In our study the participants were up to 17 years. However, no association between age and AHA-units was found in the current study (rs =.202, p=.576), nor in a previous study by our research group [8] with similar age range. The Baseline and Post 2 months AHA-unit scores for the participants were compared to blinded scorings made by an occupational therapist not involved in the treatment or data collection. The intra-class correlation between the two sets of scorings was high (ICC = 0.89).

Neuromuscular body functions were evaluated by testing active range of motion, muscle strength and muscle tone in spastic muscles. Active range of motion of the elbow and forearm was measured using a mechanical goniometer following standard procedures [34]. A stationary dynamometer BIODEX System 3 Pro (Biodex Medical Systems, Shirley, NY, USA), found to be reliable in the lower extremities of children with CP [35,36], was used to evaluate muscle tone and strength in the elbow and forearm. Measurements in the elbow were performed with the forearm in neutral position and those in the forearm with the elbow in 90° flexion. The shoulder was slightly abducted in all measurements. Muscle tone was evaluated as resistance to passive movement during three trials at two velocities, 10°/s and 180°/s, in elbow extension and forearm supination. Strength was evaluated during three dynamic maximal voluntary contractions (MVC) at a velocity of 60°/s. One practice trial was performed first to ensure that the task was performed correctly. There was a one-minute break between the trials. The procedure was first carried out on the forearm, then on the elbow. Data analysis was carried out using Matlab (The Mathworks Inc., Natick, MA, USA), version 7.6. Prior to further analyses, torque signals were low-pass filtered with a cut-off frequency of 6 Hz. The torque recorded during the passive movements at 10°/s was used to correct the other torque measures for arm weight. The peak resistance torque during 180°/s in the trial with the least resistance was used to reflect muscle tone in the elbow flexors and forearm pronators [35]. The highest peak torque value during the three isokinetic contractions was used as strength parameter.

Isometric grip force was measured using Grippit® (AB Detektor, Göteborg) in the standardized position recommended by the American Society of Hand Therapists for hand-grip dynamometry [37]. Grippit has been found to be a reliable instrument for measuring peak grip strength in children of different age groups [38]. Three maximal trials were performed for each hand, with a two-minute rest period between repetitions. The highest peak value during the trials was used for further analyses.

### Statistical analysis

Data were analysed using SPSS, version 18.0 (SPSS Inc., Chicago, IL, USA). Non-parametrical analyses were applied because of the small sample size and deviation from a normal distribution for several of the variables. Within-group differences were tested using Wilcoxon Signed Rank Test, and between-group differences by means of Mann–Whitney U test for Baseline and in change scores between Baseline to Post 2 and Post 5 months, respectively. Post 2 months and Post 5 months were tested separately against Baseline in order to reduce the effect of missing data. Significance level was set at *p* < .05 and trends at *p* < .1 are reported. Post-hoc power calculation suggested that given the sample size, our study had 80% power to detect a difference in AHA of three units from Baseline to Post-intervention with a *p*-value < .05.

## Results

All participants completed the intervention period and the subsequent Post 2 months assessment. One participant in group BT dropped out of the Post 5 months assessment.

At Baseline, the only significant group difference was higher forearm supination resistance torque in group B than in group BT (*p* = .05). Despite differences in gender distribution between the groups, there were no differences for any of the strength parameters at baseline.

**Figure 1:**
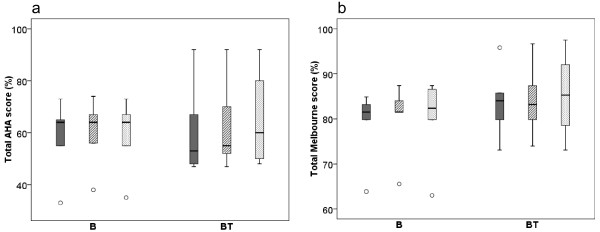
**Hand and arm use.** Box-plots for hand and arm use measured with the Assisting Hand Assessment (AHA) (**a**) and the Melbourne Assessment for Unilateral Limb Function (**b**). Baseline Post 2 months Post 5 months measures’ with Baseline (solid/dark grey) Post 2 months (striped grey) Post 5 months (dotted/light grey) measures for group B (receiving Botulinum Toxin-A) and group BT (receiving Botulinum Toxin-A + 8 weeks of resistance training). Boxes are inter-quartile ranges, the solid horizontal line is the median, whisker bars are the extreme values. Outliers are shown as circles.

Results for the hand and arm use measures at Baseline, Post 2 and Post 5 months are shown in Figure 1. Both groups had small improvements in hand and arm use. However, none of the changes were statistically or clinically significant. For group B, the median change score from Baseline to Post 2 and Post 5 months was 1 AHA-unit, while the results for group BT were 1 and 0.5 AHA-unit respectively. The equivalent results from Melbourne for group B were 1.7 and 0.8% change, and for group BT 0.8 and 0.4% change. There were no between-group differences.

**Figure 2:**
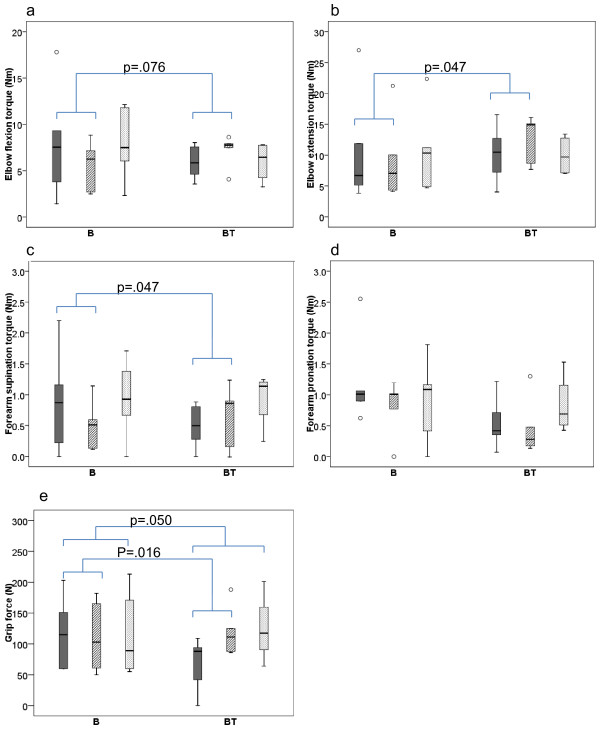
**Strength measures.** Box-plots for elbow flexion (**a**) and extension (**b**), and forearm supination (**c**) and pronation (**d**) voluntary peak torque in Newtonmeter (Nm), and grip force (**e**) in Newton (N). Baseline Post 2 months Post 5 months measures’ with Baseline (solid/dark grey) Post 2 months (striped grey) Post 5 months (dotted/light grey) measures for group B (receiving Botulinum Toxin-A) and group BT (receiving Botulinum Toxin-A + 8 weeks of resistance training). Boxes are inter-quartile ranges, the solid horizontal line is the median, whisker bars are the extreme values. Outliers are shown as circles. Significant group differences in treatment effect are indicated.

Findings for the strength measures at Baseline, Post 2 and Post 5 months are shown in Figure 2. All participants in group B lost pronation strength (*p* = .043), and the majority also experienced small, non-significant strength losses for all other strength parameters (elbow flexors: n = 4, elbow extensors: n = 3, supinators: n = 4, and grip strength: n = 3). In group BT, all participants increased their grip strength (*p* = .043). Otherwise there were no significant changes, although most children (n = 4) gained strength in elbow flexors, elbow extensors, and supinators, whereas pronation strength decreased in 3 children. When comparing the groups, there were significant or close to significant differences in treatment effects from Baseline to Post 2 months for all strength parameters (*p*’s = .016–.076) except for pronation strength. From Baseline to Post 5 months, the only significant difference in treatment effect between the groups was in grip strength (*p* = .050). Otherwise, both groups were more or less back at their Baseline values.

**Figure 3:**
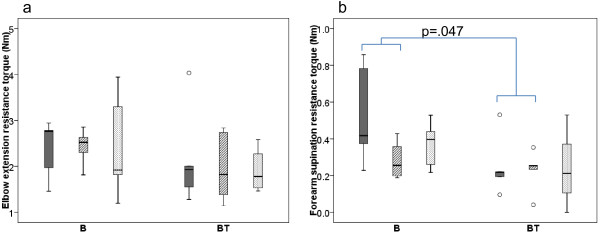
**Muscle tone measures.** Box-plots for elbow extension resistance torque and forearm supination resistance torque in Newtonmeter (Nm). Baseline Post 2 months Post 5 months measures’ with Baseline (solid/dark grey) Post 2 months (striped grey) Post 5 months (dotted/light grey) measures for group B (receiving Botulinum Toxin-A) and group BT (receiving Botulinum Toxin-A + 8 weeks of resistance training). Boxes are inter-quartile ranges, the solid horizontal line is the median, whisker bars are the extreme values. Outliers are shown as circles. Significant group differences in treatment effect are indicated.

The muscle tone results, measured as resistance to passive elbow extension and forearm supination, are shown in Figure 3. In both groups there was only a small, non-significant decrease or no change in elbow flexor muscle tone from Baseline to Post 2 and Post 5 months, with no between-group differences in treatment effect. Forearm supination resistance decreased in both groups, but considerably more so in group B (*p* = .043), resulting in a significant difference in treatment effect between the groups (*p* = .047).

**Figure 4:**
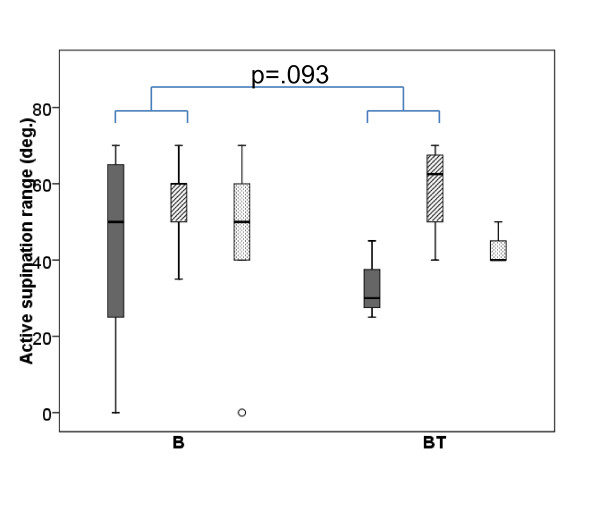
**Forearm supination.** Box-plot for active forearm supination range in degrees (deg.). Baseline Post 2 months Post 5 months measures’ with Baseline (solid/dark grey) Post 2 months (striped grey) Post 5 months (dotted/light grey) measures for group B (receiving Botulinum Toxin-A) and group BT (receiving Botulinum Toxin-A + 8 weeks of resistance training). Boxes are inter-quartile ranges, the solid horizontal line is the median, whisker bars are the extreme values. Outliers are shown as circles. Significant group differences in treatment effect are indicated.

The results from active forearm supination range at Baseline, Post 2 and Post 5 months are shown in Figure 4. All participants in group BT improved their supination range with a median change of 27.5° (*p* = .043 from Baseline to Post 2) and 10° (*p* = .066 from Baseline to Post 5). For group B, the results were less consistent. At both post-tests, two children showed improved range, two had not changed, and one showed decreased range, giving a median change in active supination range of 0°. Comparing the two groups, there was a statistical trend in favour of group BT (*p* = .093) for improvements in active supination range from Baseline to Post 2 months, but no difference between the groups at 5 months. Nearly all participants had close to full active elbow extension range at Baseline, which did not change following intervention.

## Discussion

This pilot study explored the effects of resistance training in addition to BoNT-A treatment on hand and arm use and neuromuscular body functions in a small sample of children with CP. Our results indicate that the addition of eight weeks resistance training strengthens non-injected muscles temporarily, and may possibly reduce the short-term strength loss that results from BoNT-A injections in the spastic muscles, without a concomitant systematic increase in muscle tone. Furthermore, additional resistance training may increase active range of motion to a larger extent than BoNT-A alone. However, none of the improvements in neuromuscular body functions further improved hand and arm use at the level of activity in ICF.

After eight weeks of intensive training, median strength improvements of 32 to 50% were observed in non-injected antagonists to the spastic muscles (range elbow extensors: −14 to 42% and range supinators: −58 to 72%). This is similar to strength improvements reported previously in the upper extremities [17,39]. However, our results demonstrate large variations in the effect of resistance training in this small sample, and some of the participants experienced strength losses despite resistance training. It can therefore be speculated that some children with CP might benefit more from resistance training than others. Based on a review of randomised controlled trials in the lower limbs, it has recently been suggested that not all children with CP respond to resistance training to the same extent [40]. Age and severity might have an effect, and the degree of selective motor control might also affect the response to training for some children with unilateral CP [40]. Larger studies are needed to further investigate this suggestion, using analytical approaches to determine the sources of variability.

BoNT-A injections alone resulted in a significant decrease in muscle tone, but also in a temporary strength loss in injected spastic muscles, similar to findings reported by others [14,17,26]. Despite decreased muscle tone, the ability to generate force in the antagonists to the injected spastic muscles did not show even a trend towards improvement in the participants receiving BoNT-A treatment only. This indicates that spastic activity does not limit strength in the antagonist to the spastic muscle, at least not at an isokinetic velocity of 60 °/sec. Additional resistance training improved muscle strength in injected agonists in three children, and it is therefore possible that strength losses associated with BoNT-A injections may be counteracted by progressive resistance training following approved guidelines. Larger clinical trials are needed to further illuminate these results, especially since several of the children in our study were still weakened in the injected muscles despite resistance training. Consequences of weakening injected muscles should therefore be considered when using BoNT-A [17]. In line with current literature [40,41], no systematic increase in muscle tone was found following resistance training.

Active supination range improved more in the group who performed resistance training in the BoNT-A effect period, and seems to be more effective than BoNT-A alone, showing a trend approaching significance. Strengthening of antagonists to spastic muscles in combination with reduction of muscle tone in spastic agonists is a recommended approach [9], but few studies have addressed the combined effect thoroughly [14]. Most studies have evaluated differences in passive range of motion following BoNT-A injections and therapy and found no effect [14]. One recent study found similar improvements in active elbow extension and wrist flexion between therapy with and without BoNT-A [17], whereas another study concerned the effect of BoNT-A in combination with therapy compared to therapy alone, and found larger improvements in active supination for therapy alone [16]. However, the latter finding could be attributed to differences between the groups at baseline. Active supination range has been found to be a significant predictor for activity in the upper extremities [8], and finding optimal interventions is thus important. It should therefore be investigated whether or not resistance training alone is more effective in improving active supination range than resistance training in combination with BoNT-A, since our results indicate that muscle weakness constrains active movement to a larger extent than spasticity.

Grip strength and muscle strength in antagonists to the spastic muscles (elbow extensors and forearm supinators) improved significantly more in the group who performed resistance training in combination with BoNT-A, but did not further improve unimanual or bimanual hand and arm use compared to BoNT-A alone. Similar results were obtained in a recent study by Rameckers and co-workers [17] showing that increased muscle strength and accuracy do not necessarily transfer to improved use of the affected hand in fine motor activities as measured with Melbourne. There might be several reasons for this lack of transfer. To begin with, our sample size is small and possibly a larger sample would have revealed other findings. However, none of the participants in our study had improvements of 5 AHA-units or more that is reported to be the smallest detectable difference to measure real change [32]. A second possibility is that strength gains in our study were not large enough to engender a meaningful change in hand and arm use, or that strength gains transfer to other functions than those measured with Melbourne and AHA. AHA is thought to reflect how a child performs in his or her usual environment [42], but no test performed in a standardized environment can fully capture how a child performs activities in daily life. Future studies should therefore include this aspect. A final explanation for the lack of carry-over to functional tasks could be specificity of training. In particular, for children who are unilaterally affected in the upper limbs, it may be due to problems with motor planning [43] and potential non-use of the affected hand [44], which could offset gains at the level of body function. This is in accordance with Damiano [45] who proposed that treating the impairment alone may be too far removed from the functional tasks we want to improve. The use of task-related resistance training following single-joint resistance training may have greater impact on the use of the affected hand in bimanual activities [10] and would possibly also have more lasting effects on muscle strength. In our study, none of the strength improvements were long-lasting. If the participants had integrated and transferred the improved muscle strength to improved hand and arm use, there would be a greater possibility for longer lasting effects also on muscle strength. Moreover, it is more likely that the improved limb functions will be implemented in activities when these activities are explicitly described as individual goals at the start of therapy.

In addition to the small sample size, the present study has some other limitations. Assignment to the different interventions was done by matching the children in pairs followed by tossing a coin, a method that has been found to be sensitive to selection bias. Nonetheless, the procedure resulted in two groups with similar baseline assessments, except for pronation resistance torque and gender. The difference in gender between the groups could have confounded the results if treatment effect would depend on gender. So far, however, no indications of such a gender effect are present in the literature, but caution is warranted. Moreover, the National Strength and Condition Association [28] states in their updated position statement paper on youth resistance training that there is no clear evidence of any major difference in strength between boys and girls in preadolescents. Thus, we consider it unlikely that gender is a confounder in this study. Although difficulty performing daily activities was not included as a criterion for inclusion, AHA and Melbourne indicated that all participants had limitations in the use of the involved hand.

## Conclusions

It has previously been found that muscle strength and active supination range are related to hand and arm use. It is therefore important to investigate whether treatments targeting these impairments at the level of body function will have an effect on the level of activity. We found that resistance training in combination with BoNT-A did not improve hand and arm use further compared to BoNT-A alone, even though grip strength and strength in antagonists to spastic muscles improved significantly more for the BT group. Specificity of training and strength gains could be the explanation for this lack of transfer. In future studies, there is a need for more insights into whether the use of more task-related resistance training, alone or in combination with single-joint resistance training, has greater impact on the use of the affected hand in bimanual activities, and results in longer-lasting strength effects than only the general resistance training employed in the current study.

Resistance training improved strength temporarily in non-injected muscles and may possibly compensate for the temporary strength loss in injected spastic muscles due to BoNT-A treatment, without a concomitant increase in muscle tone. However, larger clinical trials are needed to further investigate whether strength losses associated with BoNT-A may be counteracted by resistance training. Furthermore, it should be investigated whether resistance training in addition to BoNT-A injections improves active range of motion to a larger degree than resistance training alone.

## Competing interests

The authors declare that they have no competing interests.

## Authors’ contributions

AKGE: substantial contributions to the conception and design, acquisition of data, analysis and interpretation of data, drafting and revising the article critically. SMB: substantial contributions to the conception and design, acquisition of data, analysis and interpretation of data, drafting and revising the article critically. RS: substantial contributions to the conception and design, acquisition of data, and revising the article critically. TL: substantial contributions to the conception and design, and revising the article critically. BV: substantial contributions to the conception and design, data analysis and interpretation of data, drafting and revising the article critically. KR: substantial contributions to the conception and design, acquisition of data, analysis and interpretation of data, drafting and revising the article critically. All authors read and approved the final manuscript.

## Pre-publication history

The pre-publication history for this paper can be accessed here:

http://www.biomedcentral.com/1471-2431/12/91/prepub
